# Assessment of the Degradation Mechanisms of Cu Electrodes during the CO_2_ Reduction Reaction

**DOI:** 10.1021/acsami.2c23007

**Published:** 2023-06-15

**Authors:** Rik V. Mom, Luis-Ernesto Sandoval-Diaz, Dunfeng Gao, Cheng-Hao Chuang, Emilia A. Carbonio, Travis E. Jones, Rosa Arrigo, Danail Ivanov, Michael Hävecker, Beatriz Roldan Cuenya, Robert Schlögl, Thomas Lunkenbein, Axel Knop-Gericke, Juan-Jesús Velasco-Vélez

**Affiliations:** † Department of Inorganic Chemistry, Fritz-Haber-Institut der Max-Planck-Gesellschaft, 14195 Berlin, Germany; ‡ Department of Interface Science, Fritz-Haber-Institute of the Max-Planck Society, 14195 Berlin, Germany; § State Key Laboratory of Catalysis, Dalian Institute of Chemical Physics, Chinese Academy of Sciences, 116023 Dalian, China; ∥ Department of Physics, Tamkang University, New Taipei City 25137, Taiwan; ⊥ Helmholtz-Zemtrum Berlin für Materialien und Energie, 14109 Berlin, Germany; # Theoretical Division, Los Alamos National Laboratory, Los Alamos, New Mexico 87545, United States; ∇ School of Sciences, University of Salford, Environment and Life, Cockcroft Building, M5 4WT Manchester, U.K; ○ Department of Heterogeneous Reactions, Max Planck Institute for Chemical Energy Conversion, 45470 Mülheim an der Ruhr, Germany; ◆ ALBA Synchrotron Light Source, Cerdanyola del Vallés (Barcelona) 08290, Spain

**Keywords:** CO_2_RR, copper degradation, *in situ* EC-SEM, *in situ* X-ray spectroscopy, long-term reactions, electrocatalysis

## Abstract

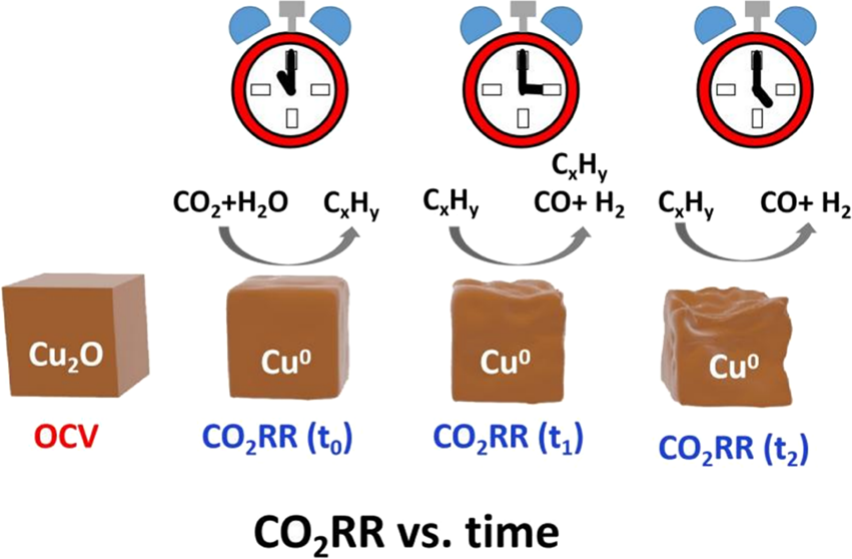

Catalyst degradation and product selectivity changes are two of the key challenges in the electrochemical reduction of CO_2_ on copper electrodes. Yet, these aspects are often overlooked. Here, we combine *in situ* X-ray spectroscopy, *in situ* electron microscopy, and *ex situ* characterization techniques to follow the long-term evolution of the catalyst morphology, electronic structure, surface composition, activity, and product selectivity of Cu nanosized crystals during the CO_2_ reduction reaction. We found no changes in the electronic structure of the electrode under cathodic potentiostatic control over time, nor was there any build-up of contaminants. In contrast, the electrode morphology is modified by prolonged CO_2_ electroreduction, which transforms the initially faceted Cu particles into a rough/rounded structure. In conjunction with these morphological changes, the current increases and the selectivity changes from value-added hydrocarbons to less valuable side reaction products, *i.e.*, hydrogen and CO. Hence, our results suggest that the stabilization of a faceted Cu morphology is pivotal for ensuring optimal long-term performance in the selective reduction of CO_2_ into hydrocarbons and oxygenated products.

## 1 Introduction

Electrochemical reduction of CO_2_ into valuable hydrocarbons and oxygenated products, powered by renewable energy, plays a key role in the solution to global warming.^1,2^ In this technology, renewable electrical energy is stored via the production of valuable chemical energy vectors from CO_2_ to build a circular economy. At present, copper is the best candidate to accomplish this reaction, because it can uniquely electroreduce CO_2_ to high-value hydrocarbons and alcohols in an aqueous electrolyte under mild conditions, as shown first by Hori and co-workers.^3,4^ However, the selective reduction of CO_2_ into fuels is very challenging because of the large number of steps involved in the reaction,^5^ steps that are yet not fully understood.^6,7^ Three major challenges are associated with the cathodic CO_2_ reduction reaction (CO_2_RR) process: (i) the relatively low faradaic efficiency due to the competition with hydrogen evolution and CO byproduct formation, (ii) high overpotentials,^8^ and (iii) the loss of selectivity for hydrocarbons and oxygenated products over time.^9,10^ The primary focus in the scientific literature is on understanding the nature of the active/selective sites^11^ as a way to aid in activity/selectivity optimization. However, preventing the loss of selectivity that Cu electrodes suffer during long-term bulk electrolysis is also a major limiting factor for the commercialization of the technology. In general, a decrease of CH_4_ and C_2_H_4_ product evolution is observed concurrently with an increase in H_2_ production through the hydrogen evolution reaction (HER), together with increased carbon monoxide formation.^12^ The observed changes in selectivity from hydrocarbons to CO and H_2_ have been ascribed in the literature to different reasons, such as (i) morphological changes in the surface of the catalyst and the concomitant presence of more kinks and defects, *i.e.*, loss of the selective lattice termination;^13−15^ (ii) poisoning effects induced by the accumulation of insoluble reaction products on the catalyst surface;^9,10^ (iii) deposited impurities from the electrolyte salts, mostly metals, which promote HER;^10^ and (iv) reduction of the electrode over time, resulting in the loss of dissolved oxygen atoms in the near surface that stabilize highly active/selective cationic copper species.^16^ Furthermore, different deactivation times and selectivity changes were reported by different groups, suggesting a strong dependence on the preparation method and experimental conditions.^17,18^

This study aims to shed light on the atomistic aspects controlling the deactivation of copper over prolonged reaction time. In order to provide detailed information about the mechanism of copper deactivation during the CO_2_RR, we monitored the evolution of the catalyst structure using *in situ* techniques under relevant reaction conditions. While these experiments remain challenging because of technical limitations—such as the need for vacuum in soft X-ray-based spectrometers and electron microscopes—we have recently developed approaches to overcome these challenges. These advances enable us to follow the evolution of the catalyst under constant potential during long-term operation.^19^ This approach allows for a direct correlation between the electronic structure and morphology modifications that the catalyst undergoes while its activity/selectivity to different reaction products changes over long-term reaction time.

## 2 Experimental Section

### 2.1 *In Situ* Electrochemical Flow Cell for X-ray Absorption Spectroscopy and Beamline

The main body of the electrochemical cell is made of polyether ether ketone (PEEK), which is electrically insulating and chemically inert. This cell has a Pt wire for a counter electrode and a Ag/AgCl reference electrode (FLEXREF, sourced from WPI Florida). This setup was designed to use an exchangeable working electrode to allow the operation of different membranes with the same EC cell. The working electrode holder is compatible with the cell body used in the environmental scanning electron microscope (ESEM), FEI Quantan 200 FEG, allowing *in situ* electrochemical microscopy characterization. For the *in situ* characterization of the electronic structure, the EC flow cell was operated in the main chamber of the ISISS beamline in BESSY II (Berlin, Germany) which is equipped with a SPECS PHOIBOS 150 NAP hemispherical analyzer. The total fluorescence yield (TFY) signal was collected with an AXUV100 Opto Diode Corp which was located in the main chamber.

The *in situ* microreactor was operated in the main chamber of the ISISS beamline in BESSY II (Berlin, Germany) with a background pressure of ∼10^–7^ mbar, while the aqueous electrolyte circulated on the back side of a Si_3_N_4_ membrane, where the electrodeposited thin-film electrodes are placed. In the ISISS beamline, the photons are sourced from a bending magnet (D41) and a plane grating monochromator (PGM) yielding an energy range from 80 to 2000 eV (soft X-ray range), a flux of 6 × 10^10^ photons/s with 0.1 A ring current using a 111 μm slit, and an 80 μm × 150 μm beamspot size. The effective area of the electrode was ∼1.5 cm^2^, which was determined by the diameter of the O-ring (0.7 cm) used for sealing the cell. The measurements were recorded at a cff of 1.4 to avoid overlapping contribution of the second order Si K-edge (from the Si_3_N_4_ membrane) on the plateau before the pre-edge of the Cu L_2,3_ edges. Note that no beam effects were observed during consecutive scans of the Cu L_2,3_-edge region, ruling out detectable beam damage in the copper electrodes. The Si_3_N_4_ membrane was used as working electrodes and X-ray windows at the same time that it separates the liquid, where the photodiode detector was placed (AXUV100 Opto Diode Corp). The X-ray transmission through the Si_3_N_4_ membrane was estimated to be approximately equal to 90% of the incoming X-ray intensity at the Cu L_2,3_-edge excitation energies. The main body of the cell was made of polyether ether ketone (PEEK), which is an electrical insulator and is chemically inert to most of the aqueous electrolytes. The counter electrode was a Pt wire and the reference electrode was a Ag/AgCl FLEXREF, sourced from WPI (Florida). A scheme of the detection approach used for in situ X-ray absorption spectroscopy- total fluorescence yield (XAS-TFY) and electrochemical scanning electron microscopy (EC-SEM) is shown in Figure S4.

### 2.2 Electrode Preparation

The pristine Si_3_N_4_ membranes (type NX10100C) were sourced from Norcada (Edmonton, Canada). This membrane is semitransparent to the incoming X-ray and separates the electrolyte from the vacuum chamber where the photodetector was located. On the Si_3_N_4_ membrane (100 nm thick), a thin film of the Cr (3 nm) adherence layer was deposited by physical vapor deposition (PVD). After that, 20 nm of Au was deposited onto the 3 nm Cr by PVD. We obtained a homogeneous polycrystalline thin film with an X-ray transmission through this membrane of approx. equal to 80% of the incoming intensity in the Cu L_2,3_-edge range. The sealing of this cell was assured with a Kalrez O-ring of 0.7 cm diameter, yielding an effective working electrode area of ∼0.4 cm^2^ at a background pressure of ∼10^–7^ mbar while the aqueous electrolyte circulated through the EC cell. The continuous flow of electrolyte helps to eliminate the bubbles produced during the electrocatalytic reactions. The copper electrode was prepared by electrodeposition from 5 mM CuSO_4_ at −0.7 V *vs* Ag/AgCl for 60 s. The electrolyte was prepared by diluting 0.798 g of CuSO_4_ (Sigma Aldrich, anhydrous powder, 99.99%) in 1 L of Milli-Q water (18.2 MΩ) at room temperature (RT), 25 °C, and saturated with pure N_2_ by gas bubbling through the electrolyte. This process yields the deposition of a copper electrode ∼300 nm thick as shown in Figure S1A. After the electrodeposition of copper, the cell was flushed with pure water and subsequently filled with 100 mM KHCO_3_, which was prepared by diluting 10 g of KHCO_3_ (Roth, 99%) in 1 L of Milli-Q water (18.2 MΩ) at room temperature and saturated with pure CO_2_ by bubbling the electrolyte. To prove the operation of this cell, Video 1 shows the CA process at −1.8 V *vs* Ag/AgCl where the formation of bubbles is because of the CO_2_RR and hydrogen evolution reaction.

### 2.3 Potentiostats

Potentiometric control during the *in situ* XAS-TFY characterization is guaranteed by a Biologic SP-300 (Seyssinet-Pariset, France), allowing for different electrochemical characterization modes such as cyclic voltammetry (CV), linear sweep voltammetry (LSV), and chronoamperometry (CA). The applied potential was controlled with an Autolab PGSTAT 204 potentiostat (Utrecht, Netherlands) during the online gas chromatography reaction product analysis.

### 2.4 CO_2_ Electroreduction Product Measurements

CO_2_ electroreduction measurements were carried out in a gas-tight glass H-cell separated by an anion exchange membrane (Selemion AMV, AGC Inc.). Both the working electrode and counter electrode compartments were filled with 40 mL of 100 mM KHCO_3_ (Honeywell, 99.95%) and purged continuously with CO_2_ (99.995%, 20 mL min^–1^). A KHCO_3_ solution was prepared with ultrapure water and further prepurified with a Chelex 100 Resin (Bio-Rad, 100–200 mesh). Prior to the measurement, the electrolyte was bubbled with CO_2_ for 30 min to remove oxygen and saturate the solution. A platinum gauze (MaTecK, 3600 mesh) was used as the counter electrode and a leak-free Ag/AgCl electrode (3.4 M KCl, Innovative Instruments, Inc.) as a reference electrode. The electrodes were electrodeposited on a gold mesh (Alfa Aesar 40931 gauze 0.064 mm, 99.9% metal basis) following the procedure described above for the electrodes prepared on the Si_3_N_4_ membrane. These electrodes were used as working electrodes and were contacted with a clamp wrapped by Kapton tape to avoid unwanted reactions. The potentials were controlled with an Autolab potentiostat (PGSTAT 204). All samples in this work were measured at a fixed potential of −1.8 V *vs* Ag/AgCl. The gas products were analyzed by online gas chromatography (GC, Agilent 7890B) every 20 min. H_2_ and hydrocarbons were separated by different columns (Molecular sieve 13X, HayeSep Q, and Carboxen-1010 PLOT) and quantified by a thermal conductivity detector (TCD) and flame ionization detector (FID).

### 2.5 Calculation of the Faradaic Efficiency of Gas Products


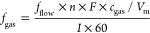
*f*_gas_: Faradaic efficiency of the gas product, %.

*f*_flow_: flow rate of CO_2_, mL min^–1^.

*I*: electrolysis current, A.

*c*_gas_: volume ratio of the gas product determined by online GC.

*V*_m_: molar volume of an ideal gas at 1 atmosphere of pressure, 22,400 mL mol^–1^.

*n*: number of transferred electrons for a certain product.

*F*: Faraday constant, 96,485 C mol^–1^.

## 3 Results and Discussion

**Figure 1:**
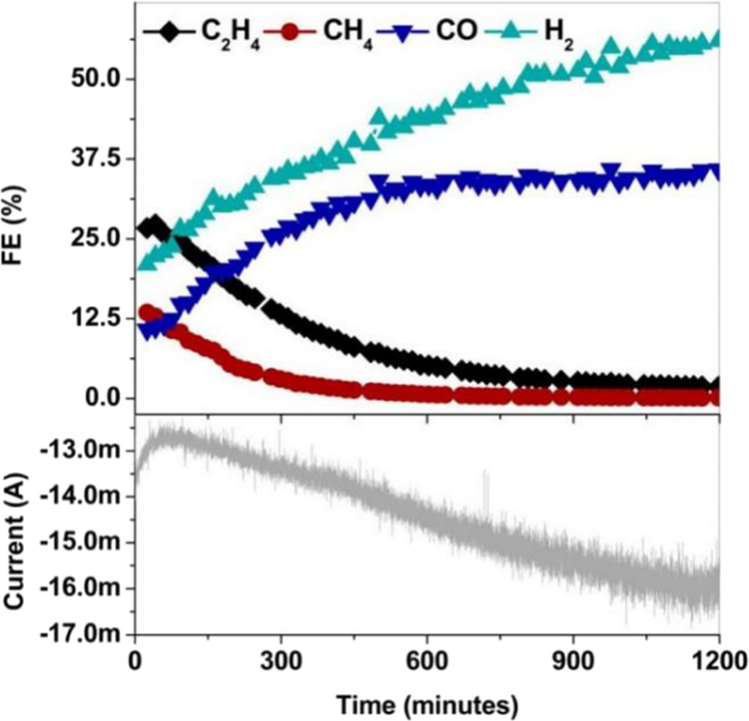
Top: gaseous product distribution of the CO_2_RR reaction (in CO_2_-saturated 100 mM KHCO_3_ at −1.8 V *vs* Ag/AgCl) over an electrodeposited copper electrode on gold as a function of time. Bottom: Total cathodic current over time collected in an H-type electrochemical cell (see the Experimental Section).

The electrode was prepared by the electrodeposition of copper on a thin-film gold electrode from a 5 mM CuSO_4_ solution, as detailed in the Experimental Section and elsewhere.^20,21^ Using this electrode, the CO_2_RR product distribution as a function of time and applied potential (−1.8 V *vs* Ag/AgCl) was investigated to provide a deeper understanding of the deactivation mechanism governing the electrode performance. Figure 1 shows the faradaic efficiency (FE) for the main gaseous products of the electroreduction of CO_2_ using the electrodeposited Cu electrode. The measurements were performed at −1.8 V *vs* Ag/AgCl in a CO_2_-saturated 100 mM KHCO_3_ electrolyte. Details of the electrochemical measurements and faradaic efficiency estimates can be found in the Experimental Section. Note that the total FE is different than 100% because the measurement does not account for the liquid products of the reaction. Figure 1 shows a change in the selectivity of the electrode over time from a multielectron transfer process leading to hydrocarbon formation towards the undesired production of CO and H_2_. Meanwhile, the total cathodic current increases over time. This is in good agreement with previous results reported in the literature.^9,10^ Several reasons have been suggested for this behavior, such as the loss of oxygen under reduction conditions, the surface accumulation of non-CO_2_RR-selective catalytic metals (contaminants), and the formation of a different interface than pure copper and/or an enhanced surface-to-bulk ratio, which allows a significantly higher amount of material to participate in the reaction.

**Figure 2:**
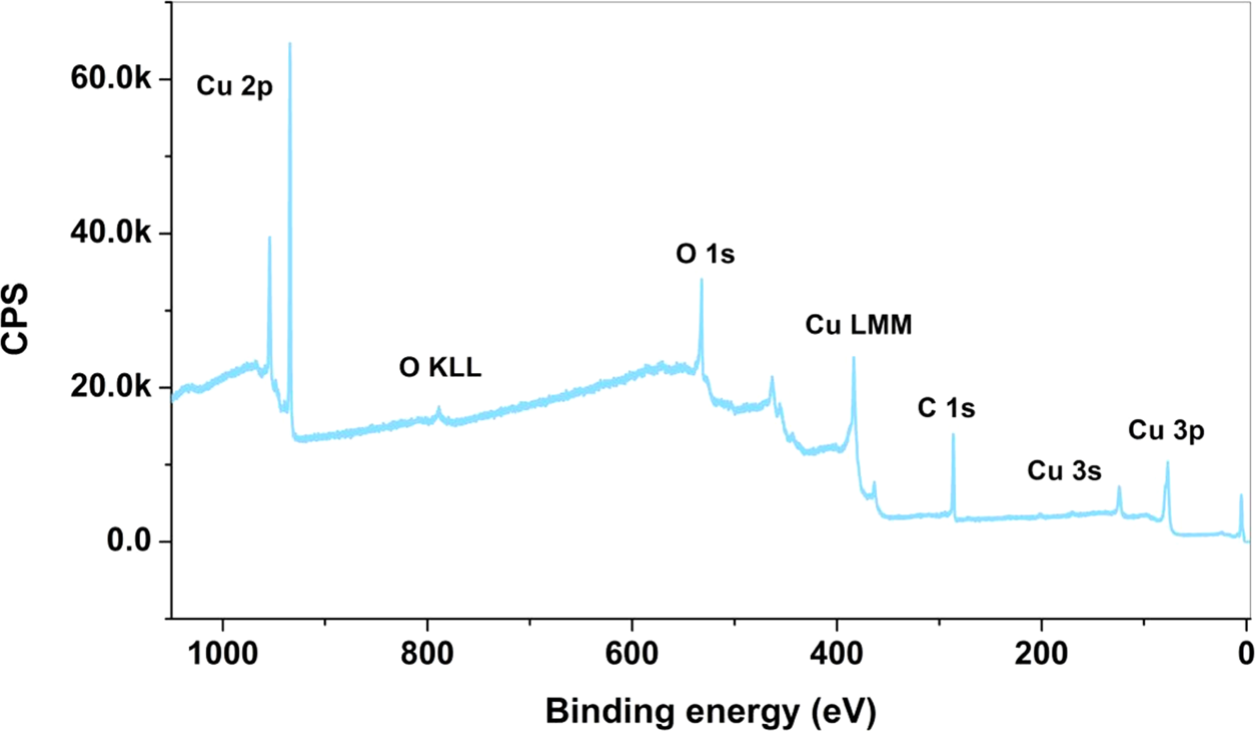
XPS survey spectra of electrodeposited Cu on an Au-coated Si_3_N_4_ membrane after the CO_2_RR.

From these possible reasons for the selectivity change, we first investigated the possibility of contamination of the electrode with other metals present in the electrolyte^9,10^ or metal cations from the counter electrode that can reach the cathode.^22^ The presence of these metals after the reaction can be determined by *ex situ* characterization using surface-sensitive X-ray photoelectron spectroscopy (XPS), which is sensitive to the presence of a sub-monolayer of foreign atoms. Such foreign metal atoms deposited on the copper electrode are not dissolved after the potential is removed in the pH 6.8 electrolyte used here, making it possible to detect them by *ex situ* XPS (within its detection limit). The absence of any impurity peaks in the survey spectra (see Figure 2, 1200 eV excitation energy) indicates that there was no evident contamination on the surface of the copper electrode with other metals over time. Also, in detailed scans with higher sensitivity (see Figure S1), no contaminations were found. Hence, the deactivation observed in Figure 1 is not related to poisoning with highly reactive materials that promote the HER.^23,24^ Furthermore, note that the C 1s spectrum indicates only the existence of a main peak at 284.4 eV, which excludes the formation of carbonates that could contribute to electrode deactivation. This is also excluded due to the fact that the electrode is not really deactivated (loss of conductivity in the isolating copper carbonates) during the long-term reaction, but rather the selectivity is changed from hydrocarbon production to CO and H_2_ formation with an enhanced cathodic current over time. Hence, we can rule out, in addition, carbon poisoning as a deactivation mechanism.

We further studied the evolution of the Cu electrocatalyst over time using *insitu* SEM and XAS. This is enabled by the modular *in situ* approach developed in our group, which is compatible with both SEM and XAS in total fluorescence yield mode (TFY-XAS). This makes it possible to correlate the changes in the product selectivity to variations in both the electronic structure and morphology under the same reaction conditions.^19^ The experimental design enables a time resolution down to a minute and allows the average information of the electronic structure collected with X-ray absorption to be compared with the local morphology changes provided by electron microscopy. Furthermore, the flow cell geometry is compatible with prolonged CO_2_RR at high current densities, in contrast to other techniques such as *in situ* transmission electron microscopy (TEM), which require microsized cells that are generally incompatible with gas evolution reactions due to bubble trapping.

**Figure 3:**
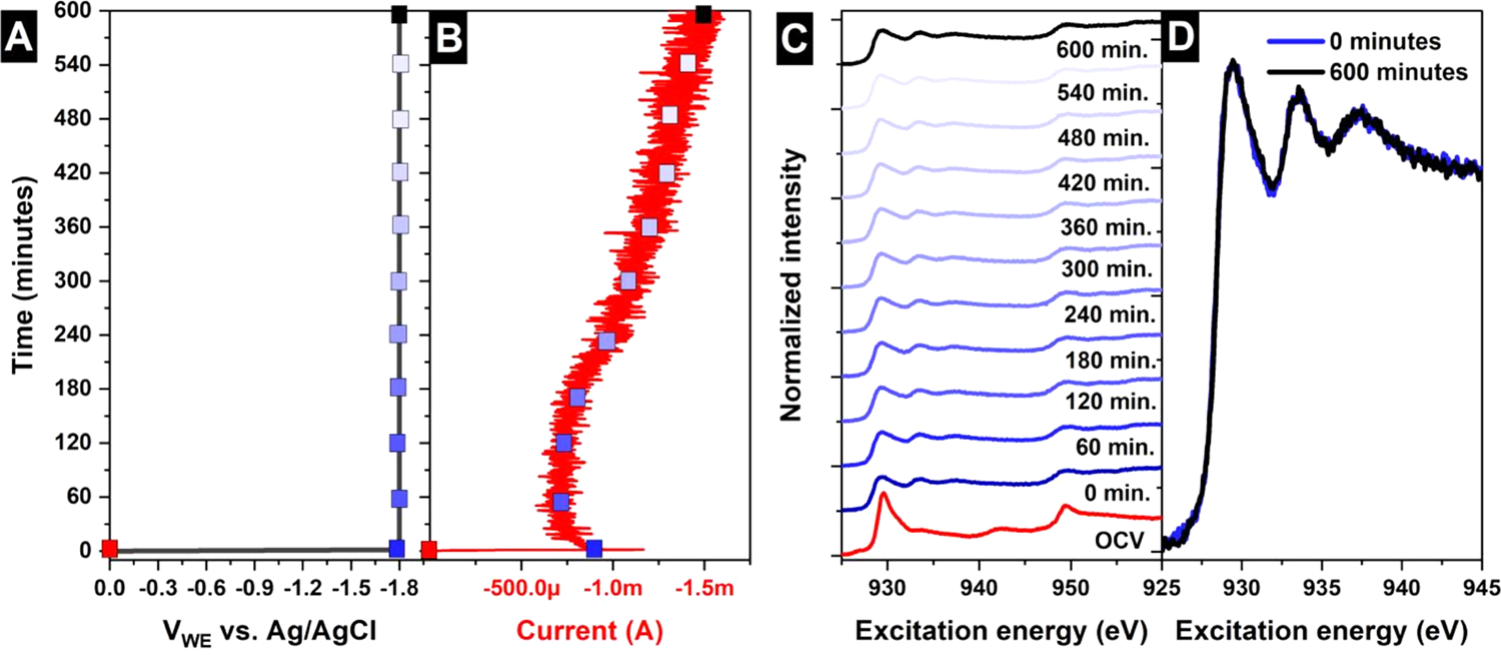
(A) Applied voltage, (B) current, and (C) Cu L_2,3_ edges in FY mode depending on the time at −1.8 V *vs* Ag/AgCl (under CO_2_RR conditions) in the presence of 100 mM KHCO_3_ saturated with CO_2_. (D) Cu L_3_ edge in FY mode depending on the time at −1.8 V *vs* Ag/AgCl (under CO_2_RR conditions) in the presence of 100 mM KHCO_3_ saturated with CO_2_.

Using *in situ* TFY-XAS, we investigated the evolution of the electrode’s electronic structure during the long-term reaction (see the Experimental Section and previous work^19,25^ for technical details). Figure 3 shows the voltage applied to the working electrode (A), current (B), and Cu L_2,3_-edge XA spectra (C) at OCV and under CO_2_RR conditions (−1.8 V *vs* Ag/AgCl) as a function of time. Analogous to the experiment in Figure 1, the cathodic current increases over time, demonstrating that the catalyst evolution is reproduced in the *in situ* experiments. Meanwhile, the Cu L_2,3_-edges indicate an immediate reduction from Cu^+^ to Cu^0^ (see Figure S2 for spectra references) upon moving from OCV to CO_2_RR conditions. Note that XAS in TFY mode detects the Cu oxidation state of both the surface and bulk, and hence the data indicate that the entire electrode is in the metallic state during CO_2_RR. No further change in the oxidation state is observed over time, which indicates that the deactivation observed is not a consequence of a variation in the oxidation state of copper or remaining trace copper oxide in the electrode bulk (see Figure 3D for a better comparison of the spectra, after 600 min of continuous CO_2_RR operation). This result proves that there is not a detectable reservoir of active oxidized species in the bulk for thin-film electrocatalysts that can diffuse to the surface and be consumed during the CO_2_RR, thereby ruling out the loss of oxygen as the reason for the low FE towards hydrocarbons after prolonged CO_2_RR. Therefore, according to our results, the surface and bulk are in the metallic stable phase in agreement with the Pourbaix diagram^26^ and previous results^19,21,27^ under CO_2_RR conditions.

**Figure 4:**
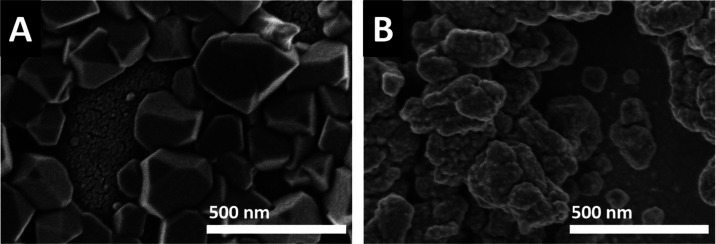
SEM images of electrodeposited copper on an Au-coated Si_3_N_4_ membrane (A) before CO_2_RR and (B) after CO_2_RR (−1.8 V *vs* Ag/AgCl in 100 mM KHCO_3_ saturated with CO_2_).

The results presented so far indicate that there was no change in the composition or electronic structure during long-term CO_2_RR. Therefore, we now focus on the morphological changes of the electrode and their link to product selectivity. Firstly, the catalyst morphology before and after prolonged CO_2_RR was investigated by *ex situ* scanning electron microscopy (SEM) in order to reveal irreversible morphological changes that could be brought about by the reaction. The comparison of the electrode before (Figure 4A) and after CO_2_RR (Figure 4B), using a secondary electron detector, indicates a significant change in the catalyst morphology before and after reactions. The initially faceted surface is transformed to a rougher one, more roundish in structure, in good agreement with measurements done with thin-film thermal oxide-derived copper after its reduction.^27^ These measurements suggest that the deactivation of the copper catalyst may be related to changes in its morphology, in line with the structure-sensitive selectivity observed for the CO_2_RR in the literature.^4,28^ However, one must note that *ex situ* microscopy characterization cannot clarify whether the morphology changes are the consequence of prolonged reaction conditions or if these changes occur immediately after starting the CO_2_RR.^29^ In addition, reoxidation of the sample after removal from the cell may have also affected the catalyst morphology. Consequently, the *ex situ* SEM characterization requires further validation by *in situ* measurements.

**Figure 5:**
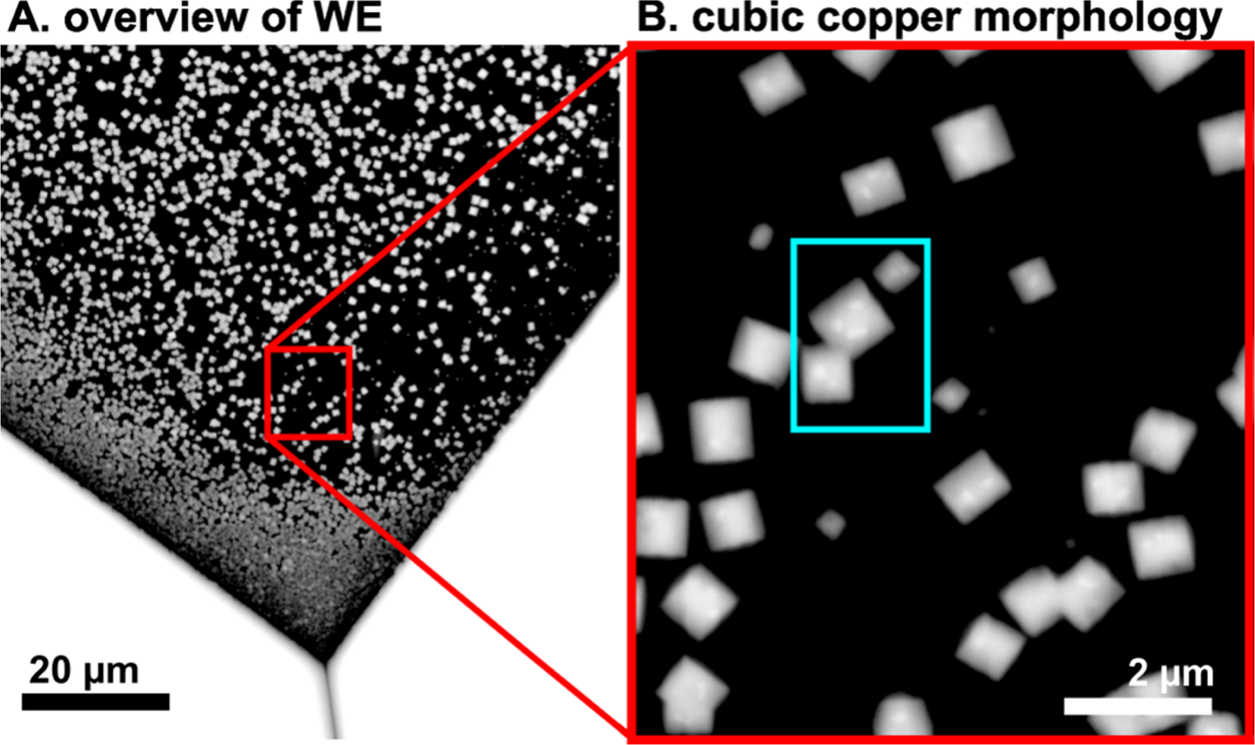
*In situ* EC-SEM images of fresh electrodeposited copper in OCV conditions using a large field: (A) overview of the transparent window exemplifying the homogeneous distribution of the material at the electrode and (B) a close-up look revealing the cubic structure of electrodeposited copper.

To characterize the morphology changes *in* situ, we performed EC-SEM measurements on cubic Cu particles shown in Figure 5. The highly faceted shape of these particles makes it possible to clearly observe morphology changes despite the lower resolution of EC-SEM compared to *ex situ* SEM. The particles were grown using a pulsed deposition procedure that was optimized *in operando* using the EC-SEM images. In the procedure, the potential was ramped from OCV into −0.7 V *vs* Ag/AgCl and left under potentiostatic reduction for 12 s. Then, the system was set back to OCV conditions for 45 s. Subsequently, the electrodeposition was initiated again by a second pulse from OCV into −0.7 V. This procedure was repeated 5 times. The pulses were aimed at stimulating the formation of discrete nucleation centers, resulting in a homogenous deposit of copper cubes evenly dispersed on the electrode. More disordered morphologies were also achieved, for instance, by keeping the electrode under polarization for 60 s (see the Supporting Material) without pulsating deposition. A video of the *in situ* pulsed deposition can be consulted in the Supporting Material (Video S1).

**Figure 6:**
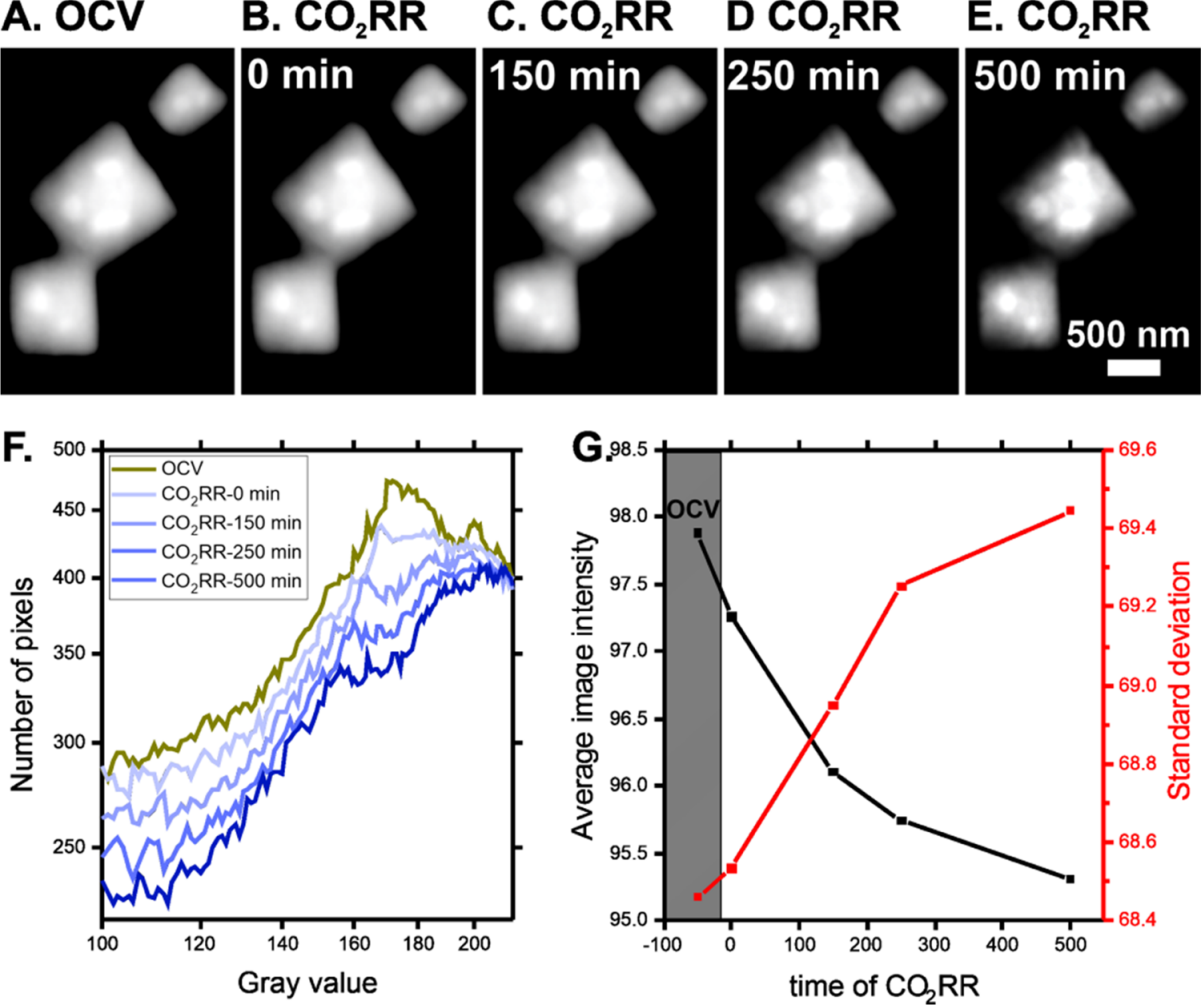
*In situ* EC-SEM of electrodeposited copper illustrating the region of interest highlighted in Figure 5B. Images were collected in 100 mM KCO_3_ (saturated in CO_2_) with Pt and Ag/AgCl as counter and reference electrodes, respectively: (A) OCV, (B) −1.8 V *vs* Ag/AgCl, (C) after 150 min at −1.8 V *vs* Ag/AgCl, (D) after 250 min at −1.8 V *vs* Ag/AgCl, and (E) after 500 min at −1.8 V *vs* Ag/AgCl. (F) Pixel distributions of the images presented in panels (A–E). (G) Average image intensities and standard deviation of the pixel distribution as a function of reaction time.

Figure 6 shows the morphological evolution of selected cubic particles (region of interest highlighted in Figure 5B) during the CO_2_RR. The starting cubes imaged under OCV (Figure 6A) exhibit regular shapes and smooth surfaces. The structure slowly evolves upon electrode polarization (Figure 6B–D), displaying particle shrinking and loss of the initially smooth morphology. These changes are subtle and occur continuously during the CO_2_RR. To assess the changes quantitatively, we calculated the pixel distributions of the images represented in Figure 6A–E. The resulting curves in Figure 6F show a gradual change in the pixel distributions over time, confirming that the evolution of the particle morphology is a long-term process. Overall, the curve becomes less intense over time and spreads toward lower picture values, as also indicated in Figure 6G. Because the brightest parts of the images compiled in Figure 6A–E correspond to the cubic copper particles, the loss of image intensities over time implies the gradual shrinking of the deposited cubes at the beginning and along the reaction. The initial shrinking at 0 min is caused by the transition from CuO*_x_* to Cu upon moving to CO_2_RR conditions (as shown in Figure 3) and is in good agreement with previous work and the literature.^19,33^ The continued shrinking of the particles during CO_2_RR suggests Cu dissolution and/or redistribution.^34^ The shrinking changes not only the size but also the shape of the particles. This is evidenced by the increase of the standard deviation of the pixel distribution (Figure 6G), which equates to a higher variation of pixel intensities. This implies a transformation from a smooth surface into a rougher morphology. Overall, these findings are in good agreement with the *ex situ* SEM analysis. EC-SEM studies on more randomly shaped particles confirmed the generality of the gradual morphology changes during CO_2_RR (see Figure S3).

From the analysis above, it is clear that the evolution of the catalyst morphology and CO_2_RR selectivity correlate, both showing gradual changes over the course of hours. This can be interpreted in terms of the structure-sensitive selectivity of the CO_2_RR on Cu. In pioneering work, Hori and co-workers have shown that the CO_2_RR selectivity of different single-crystal facets displays marked differences.^4^ Hence, catalysts with a different balance of terrace, step, and kink sites will show a different selectivity. Although SEM does not have sufficient resolution to resolve these sites, we can make a very rough estimate for our catalyst based on the particle shape. At the start of the CO_2_RR, our particles are highly faceted. Such faceted structures form to minimize the surface energy of the particles (as described by the Wulff construction). Since the surface energy of low-index facets with mainly terrace sites is the lowest, we can conclude that the particles at the start of the reaction contained a relatively large amount of terrace sites. In the rounder particle shape that evolves over time during the CO_2_RR, there is a wider variety of surface orientations. This means that the dominance of the low-index facets is lost, and hence terrace sites are lost in favor of step and kink sites. This is in line with recent *in situ* electrochemical atomic force microscopy studies.^31,32^ Since step and kink sites display a high selectivity for H_2_ and CO,^30^ it follows that the morphology changes from a faceted to a rounded particle shape are detrimental to the CO_2_RR selectivity toward the desired hydrocarbons.

## 4 Conclusions

In summary, the combination of *in situ* TFY-XAS, XPS, EC-SEM, and GC product analysis provided direct evidence of the mechanism for the selectivity loss for hydrocarbons during the CO_2_ reduction reaction on Cu particles. We have assessed the possibility of metal poisoning, depletion of trace copper oxide promotors, CuCO_3_ formation, and particle morphology changes as the origin of the selectivity loss. Our results indicate that only Cu particle morphology changes play a significant role under the employed conditions. The initially faceted Cu nanoparticles evolve in a more rounded shape, which coincides with a gradual shift in selectivity from hydrocarbons to H_2_ and CO. We attribute this correlation between the evolution of the particle shape and selectivity to the increase in the abundance of step and kink sites in a more rounded particle shape, which is known to be more selective toward H_2_ and CO. Thus, our works show that stabilizing faceted Cu particle shapes is a key factor for developing stable CO_2_RR catalysts with long-term selectivity towards hydrocarbons.

## Supporting Information Available

*Ex situ* XPS and XAS characterization (Figure S1); Cu L_2,3_-edge XAS references (Figure S2); *in situ* SEM experiment (Figure S3), and detection scheme (Figure S4) (PDF)
*In situ* pulsed deposition can be consulted (Video S1) (AVI)
CA process at −1.8 V *vs* Ag/AgCl where the formation of bubbles is because of the CO_2_RR and hydrogen evolution reaction (Video 1) (MP4)

## Supplementary Material

am2c23007_si_001.pdf

am2c23007_si_002.avi
