# Supplementary material for: Assessment of the Degradation Mechanisms of Cu Electrodes during the CO2 Reduction Reaction
Source: ACS Appl Mater Interfaces. 2023 Jun 15;15(25):30052–9. doi: 10.1021/acsami.2c23007 (PMC10316322; doi:10.1021/acsami.2c23007)
Supplement: Supplementary file 1 — am2c23007_si_001.pdf [file am2c23007_si_001.pdf]

Supplementary information:

Assessment of the degradation mechanisms of Cu electrodes during the CO_2 reduction reaction

Rik V. Mom[1, ‡], Luis-Ernesto Sandoval-Diaz[1, ‡], Dunfeng Gao[2,3], Cheng-Hao Chuang[4], Emilia A. Carbonio[1,5], Travis E. Jones[1,6], Rosa Arrigo[7], Danail Ivanov[1], Michael Hävecker[1,8], Beatriz Roldan Cuenya[2], Robert Schlögl[1,8], Thomas Lunkenbein[1], Axel Knop-Gericke[1,8], Juan-Jesús Velasco-Vélez[1,8,9]*

*Corresponding authors: velasco@fhi-berlin.mpg.de, jvelasco@cells.es

‡equal contribution

[1]Department of Inorganic Chemistry, Fritz-Haber-Institut der Max-Planck-Gesellschaft, 14195 Berlin, Germany.
[2]Department of Interface Science, Fritz-Haber-Institute of the Max-Planck Society, 14195 Berlin, Germany.
[3]State Key Laboratory of Catalysis, Dalian Institute of Chemical Physics, Chinese Academy of Sciences, 116023 Dalian, China.
[4]Department of Physics, Tamkang University, New Taipei City 25137, Taiwan.
[5]Helmholtz-Zemtrum Berlin für Materialien und Energie, 14109 Berlin, Germany.
[6]Theoretical Division, Los Alamos National Laboratory, Los Alamos, New Mexico, 87545, United States
[7]School of Sciences, University of Salford, Environment and Life, Cockcroft building, M5 4WT, Manchester, U.K.
[8]Department of Heterogeneous Reactions, Max Planck Institute for Chemical Energy Conversion, 45470 Mülheim an der Ruhr, Germany.
[9]ALBA Synchrotron Light Source, Cerdanyola del Vallés (Barcelona) 08290, Spain.

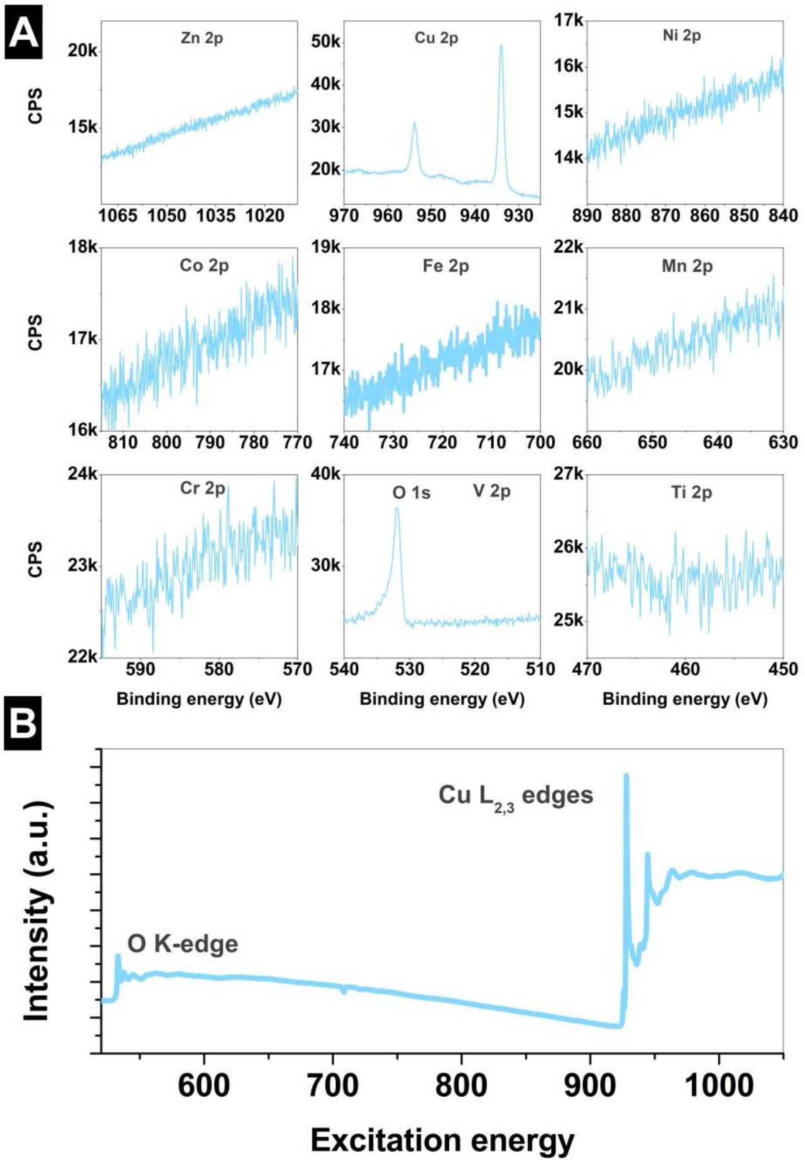

Figure S1: A XPS of selected spectra of the electrodeposited Cu on an Au-coated Si_3N_4 membrane and (100 eV kinetic energy) **B** TEY-XAS after the CO_2RR.

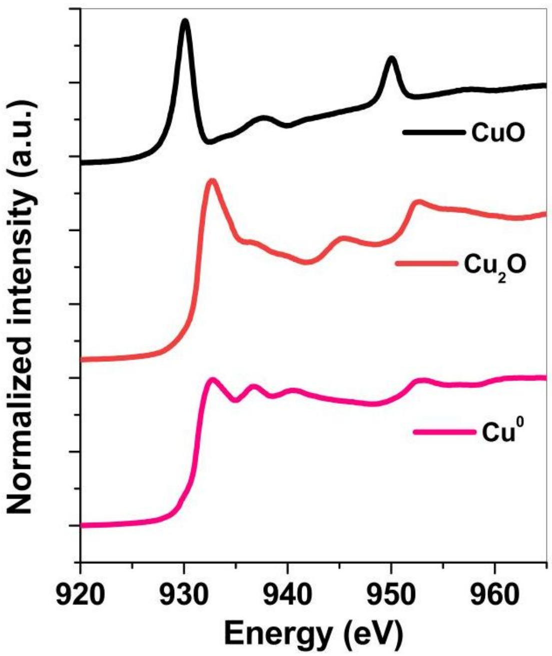

Figure S2: Cu $L_{2,3}$ edges reference spectra collected in TFY for CuO (Cu^{2+}), Cu_2O (Cu^+) and copper metal (Cu^0).

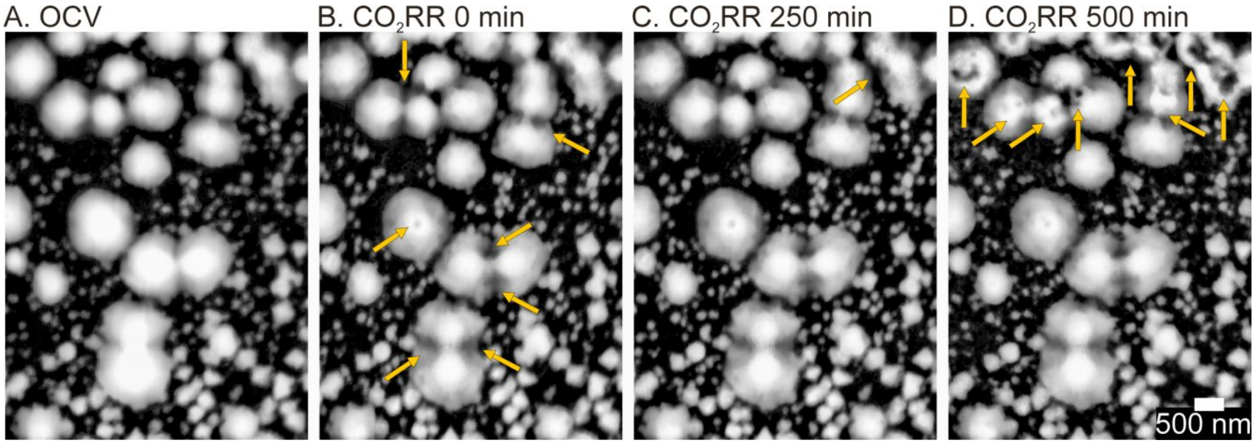

Figure S3: *In situ* EC-SEM using back scattered electrons detection mode collected in 100 mM KCO_3 (saturated in CO_2) with Pt and Ag/AgCl as counter and reference electrodes respectively: **A** OCV, **B** -1.8 V vs. Ag/AgCl, **C** after 250 minutes at -1.8 V vs. Ag/AgCl and **D** after 500 minutes at -1.8 V vs. Ag/AgCl.

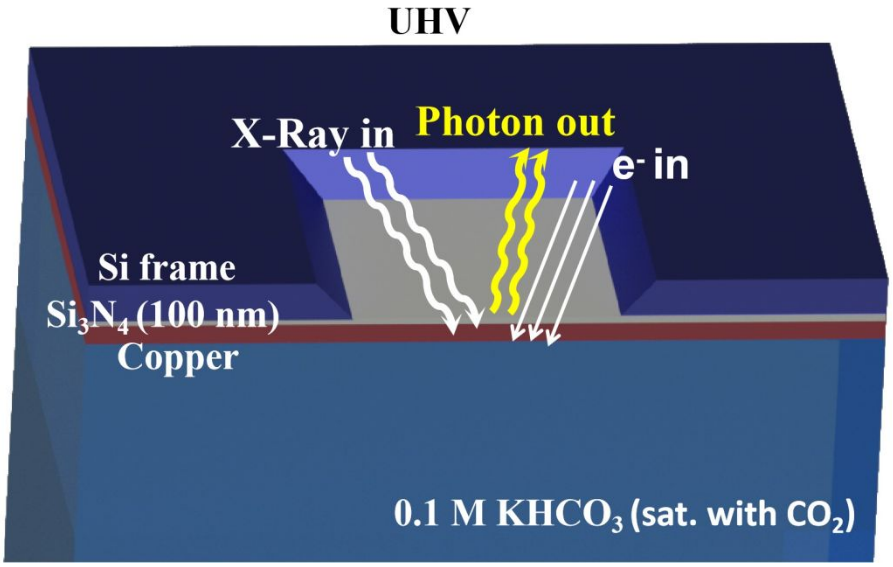

Figure S4: Scheme of the *in situ* experiment approach.
